# Supplementary material for: Knowledge, attitude, and practice regarding venous thromboembolism prophylaxis: A multicenter cross-sectional study of medical staff in Guinea
Source: PLoS One. 2025 Apr 30;20(4):e0319855. doi: 10.1371/journal.pone.0319855 (PMC12043158; doi:10.1371/journal.pone.0319855)
Supplement: S1 Appendix — (DOCX) [file pone.0319855.s001.docx]

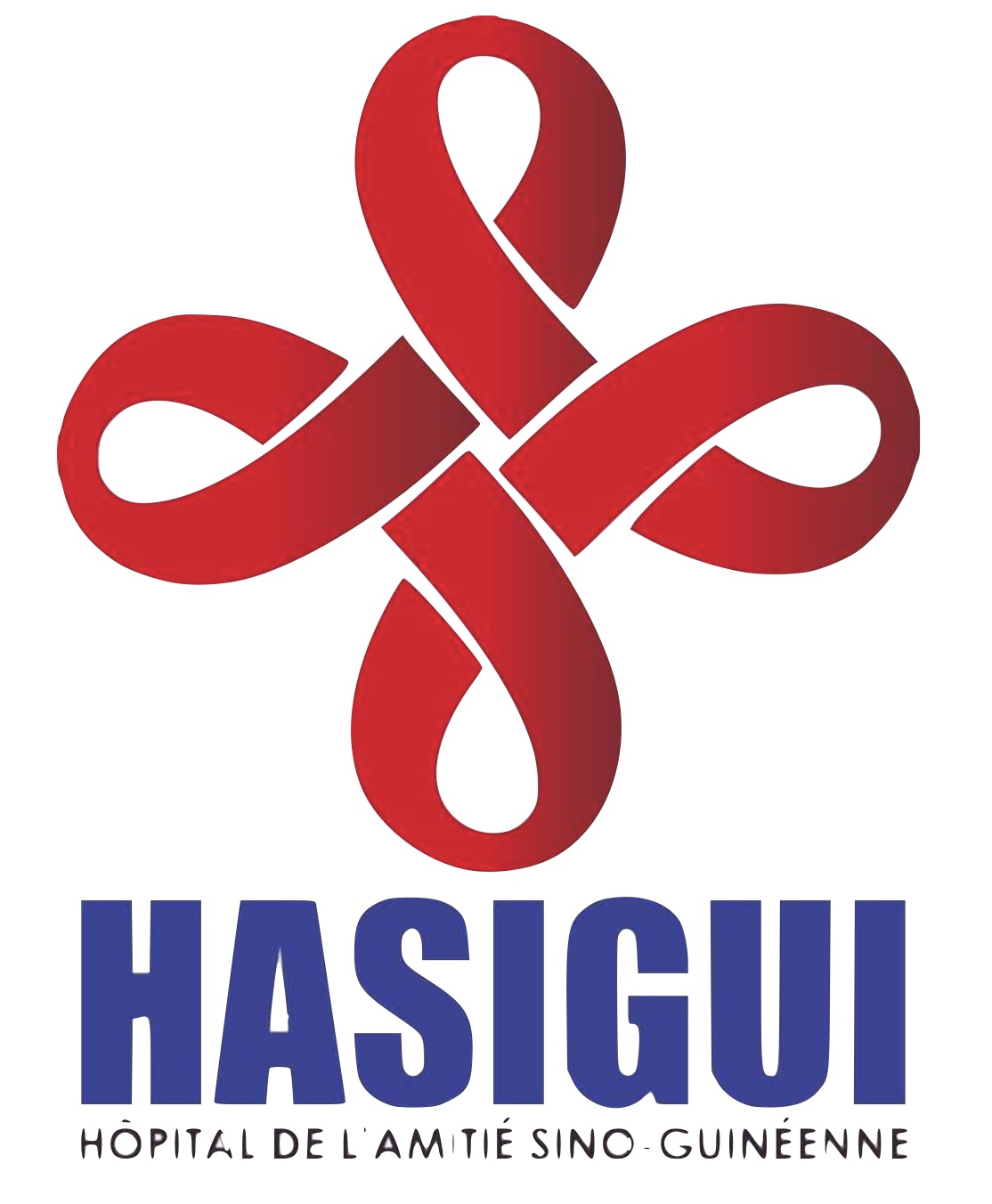
Survey

on knowledge, attitudes and practices concerning venous thromboembolism prophylaxis

with medical staff in Guinea

To find out how much you know about preventing venous thromboembolism, we invite you to take the survey. He will need your help for 15 minutes. We will respect the confidentiality of the information contained in your survey.

If you have any questions, please call Dr. Gérard 611 429 310 or

Dr. Justine 621 721 878.

Thank you very much for your cooperation!

1. **Basic information**

| **1** | **First and last name** |  |
| --- | --- | --- |
| **2** | **Age** | （ ）ans |
| **3** | **Type** | 1. man 2. woman |
| **4** | **Phone number** |  |
| **5** | **Job category** | 1. Doctor 2. nurse 3.other （ ） |
| **6** | **Service** | 1. cardiology 2. Neurology 3. Neurosurgery 4.Traumatology  5. Visceral Surgery 6. Urology 7. Thoracic Surgery  8. Acupuncture/KINE 9. Operating theatres  10. Anesthesia/Resuscitation 11. Emergencies 12. |
| **7** | **Working hours** | （ ）Year |
| **8** | **Education level** | 1.BEPC 2.BAC 3.LICENCE 4.MASTER  5.DOCTORATE 6.SPECIALIST 7.OTHER |

1. **Knowledge of venous thromboembolism prevention**

| **Basic knowledge** | True or False |
| --- | --- |
| 1. Deep vein thrombosis (DVT) is a clinical manifestation of venous thromboembolism (VTE). |  |
| 2. Pulmonary embolism (PE) is a clinical manifestation of VTE. |  |
| 3. PE is the most serious complication of DVT. |  |
| 4. Decreased blood flow velocity is one of the three main causes of VTE formation. |  |
| 5. Hyperlipidemia is one of the three main causes of VTE formation. |  |
| 6. An elevated blood coagulation state is one of the 3 main causes of VTE formation. |  |
| 7. Blood vessel damage is one of the 3 main causes of VTE formation. |  |
| 8. Redness, swelling, warmth and pain in the limbs are clinical manifestations of acute DVT. |  |
| 9. Dyspnea is a clinical manifestation of acute DVT. |  |
| 10. Chest pain is not a clinical manifestation of acute DVT. |  |
| 11. Chest pain is a clinical manifestation of acute PE. |  |
| 12. Dyspnea is a clinical manifestation of acute PE. |  |
| 13. Syncope is a major clinical manifestation of acute large-area PE. |  |
| **Risk assessment** | True or False |
| 14. Age is a risk factor for VTE. |  |
| 15. A history of malignancy is a risk factor for VTE. |  |
| 16. Inflammatory bowel disease is a risk factor for VTE. |  |
| 17. Leg swelling is a risk factor for VTE. |  |
| 18. Chemotherapy is a risk factor for VTE. |  |
| 19. Acute myocardial infarction is a risk factor for VTE. |  |
| 20. Congestive heart failure is a risk factor for VTE. |  |
| 21. Putting a cast or splint on the leg is a risk factor for VTE. |  |
| 22. Stroke (<1 month) is a risk factor for VTE. |  |
| 23. Multiple trauma (<1 month) is a risk factor for VTE. |  |
| 24. Acute spinal cord injury with paralysis (<1 month) is a risk factor for VTE. |  |
| 25. A hip, pelvis or leg fracture (<1 month) is a risk factor for VTE. |  |
| 26. Heparin-induced thrombocytopenia is a risk factor for VTE. |  |
| 27. Family history of DVT/PE is a risk factor for VTE. |  |
| 28. A history of DVT/PE is a risk factor for VTE. |  |
| 29. An existing tumor is a risk factor for VTE. |  |
| 30. Pregnancy or post-partum of less than one month is a risk factor for VTE. |  |
| 31. Stillbirth or miscarriage (more than 3) is a risk factor for VTE. |  |
| 32. Sepsis (<1 month) is a risk factor for VTE. |  |
| 33. Severe lung disease, including pneumonia (<1 month), is a risk factor for VTE. |  |
| 34. Pulmonary dysfunction (e.g. COPD) is a risk factor for VTE. |  |
| 35. Bed rest is a risk factor for VTE. |  |
| 36. Varicose veins are not a risk factor for VTE. |  |
| 37. The use of oral contraceptives is not a risk factor for VTE. |  |
| 38. Hormone therapy is not a risk factor for VTE. |  |
| 39. Obesity is not a risk factor for VTE. |  |
| 40. Central venous catheterization is not a risk factor for VTE. |  |
| **Basic prophylaxis** | True or False |
| 45. It is useful to teach bedridden patients lower limb exercises to prevent VTE. |  |
| 46. Regular movement of bedridden patients is useful in preventing VTE. |  |
| 47. Adequate hydration can prevent the onset of VTE. |  |
| 48. Smoking and alcohol cessation have no effect on VTE prevention. |  |
| 49. Controlling blood glucose and lipids can help prevent VTE. |  |
| 50. Helping hospitalized patients to perform out-of-bed activities as soon as their condition permits does not prevent VTE. |  |
| **Physical prophylaxis** | True or False |
| 51. Compression stockings can be removed at night to relax the muscles, with no effect on VTE prevention. |  |
| 52. Compression stockings must be regularly exposed to the sun to be sterilized. |  |
| 53. An alkaline detergent should be used to clean compression stockings. |  |
| 54. If compression stockings are too long, they can be folded up the thigh. |  |
| 55. The correct way to put on compression stockings is to roll them progressively from the distal to the proximal limb. |  |
| 56. Compression stockings must not be worn folded. |  |
| 58. Wearing compression stockings means that pressure sores can be ignored, because of their low incidence. |  |
| 59. The choice of compression stocking size must be made with precision. |  |
| 60. Compression stockings cannot continue to be applied when a patient has a VTE. |  |
| 61. Intermittent pneumatic compression devices can be used after a VTE has occurred. |  |
| 62. Intermittent pneumatic compression devices should be used 24 hours a day for bedridden patients. |  |
| 63. It is not important to be alert to complications while using compression stockings or intermittent pneumatic compression devices. |  |
| **Pharmacological prophylaxis** | True or False |
| 64. The correct needle puncture angle for subcutaneous injection of anticoagulants is 90 degrees. |  |
| 65. Before injecting pre-filled anticoagulants, the air contained in the needle tube must be evacuated. |  |
| 66. The most common injection site for anticoagulants is the umbilicus. |  |
| 67. Hemorrhage is the most frequent complication following anticoagulant therapy. |  |
| 68. Allergic reactions are the most frequent complication of anticoagulant therapy. |  |

1. **Attitudes and practices regarding venous thromboembolism prophylaxis**

| **Attitudes（Do you agree with the following points of view?）** | very much agree | Agreed | neutral | disagree | strongly disagree |
| --- | --- | --- | --- | --- | --- |
| 1. The risk of VTE must be assessed in hospitalized patients. |  |  |  |  |  |
| 2. A specialist physician must provide treatment for patients with VTE. |  |  |  |  |  |
| 3. A multidisciplinary team must ensure the treatment of patients with VTE. |  |  |  |  |  |
| 4. Staff must be regularly trained in VTE prophylaxis. |  |  |  |  |  |
| 5. VTE prophylaxis can improve the quality of medical care. |  |  |  |  |  |
| 6. Your medical service encourages you to find out more about VTE prophylaxis. |  |  |  |  |  |
| 7. Your hospital pays great attention to VTE prophylaxis. |  |  |  |  |  |
| 8. What concerns do you have about VTE prophylaxis? |  |  |  |  |  |
| Financial penalty if patient cannot be treated with VTE prophylaxis |  |  |  |  |  |
| Increased workload |  |  |  |  |  |
| Rising medical costs |  |  |  |  |  |
| Extended hospital stay |  |  |  |  |  |
| Exacerbation of doctor-patient conflicts |  |  |  |  |  |
| 9. What are the difficulties associated with VTE prophylaxis? |  |  |  |  |  |
| Employee knowledge and participation |  |  |  |  |  |
| Patient compliance |  |  |  |  |  |
| Cooperation between departments |  |  |  |  |  |
| Specialist's ability to treat VTE |  |  |  |  |  |
| Medical costs |  |  |  |  |  |
| **Practical（Do you meet the following requirements?）** | very much agree | Agreed | neutral | disagree | strongly disagree |
| 1. You always assess the risk of VTE in hospitalized patients. |  |  |  |  |  |
| 2. You always provide health education about VTE prophylaxis to hospitalized patients. |  |  |  |  |  |
| 3. You can give advice to patients suffering from VTE. |  |  |  |  |  |
| 4. You understand and master VTE risk assessment scales. |  |  |  |  |  |
