# Supplementary material for: Knowledge, attitude, and practice regarding venous thromboembolism prophylaxis: A multicenter cross-sectional study of medical staff in Guinea
Source: PLoS One. 2025 Apr 30;20(4):e0319855. doi: 10.1371/journal.pone.0319855 (PMC12043158; doi:10.1371/journal.pone.0319855)
Supplement: Attachment — Submitted filename: Response to Reviewers 1.6.docx [file pone.0319855.s006.docx]

Response to reviewers

Reviewer #3: thanks for the revision and address the all comments. Happy to be part to review the paper.

Response: Thank you very much for your suggestions.

Reviewer #4: The study investigated the awareness of medical staff regarding venous thromboembolism (VTE) prophylaxis in Guinea. I recommend the following minor corrections:
1. Provide more details on how you determined the sample size of 245 cases. This could include the statistical methods or calculations used, any assumptions made, and the confidence level and margin of error considered.

Response: Thank you for your suggestion. The survey was conducted in all clinical departments of the three national hospitals, the names of the included departments have been described in the methodology section, and the 245 participants included all health care workers in the above departments. We surveyed all healthcare professionals in the above departments, totaling 245. It was not a sample survey. According to your suggestion, we have clearly described the inclusion and exclusion criteria of our study.

Thank you very much.
2. Was the internal consistency of each part of the questionnaire assessed? Please report the Cronbach's Alpha value or any other relevant statistic that would help to understand the reliability of the questionnaire.

Response: Thank you for your suggestions. According to your suggestion, we have analyzed the internal consistency of the questionnaire using the Cronbach's alpha analysis. The result showed that the Cronbach's alpha value of the questionnaire was 0.92. and the Cronbach's alpha analysis has been described in our manuscript.

Thank you very much.
3. Clearly state the inclusion and exclusion criteria used in the study. This will help readers understand the characteristics of the study population and ensure the study's reproducibility and validity.

Response: Thank you for your suggestion. According to your suggestion, we have described the inclusion and exclusion criteria of our study as following.

Inclusion criteria: The participants included all the clinicians, nurses, and pharmacists from all the clinical departments including internal medicine (Cardiology, Neurology, Acupuncture, Gastroenterology, Endocrinology, Respiratory medicine, and Nephrology), surgery (Neurosurgery, General surgery, Urology, Thoracic surgery, and Operating room), Intensive Care Unit （ICU）, and Emergencies.

Exclusion criteria: Administrators, Laboratory Physicians, Imaging Physicians, and Logistics Department staff.

Thank you very much.
